# Gene deleted live attenuated *Leishmania* vaccine candidates against visceral leishmaniasis elicit pro-inflammatory cytokines response in human PBMCs

**DOI:** 10.1038/srep33059

**Published:** 2016-09-14

**Authors:** Kumar Avishek, Himanshu Kaushal, Sreenivas Gannavaram, Ranadhir Dey, Angamuthu Selvapandiyan, V. Ramesh, Narender Singh Negi, Uma S. Dubey, Hira L. Nakhasi, Poonam Salotra

**Affiliations:** 1 National Institute of Pathology (ICMR), Safdarjung Hospital Campus, New Delhi, India; 2 Division of Emerging and Transfusion Transmitted Diseases, CBER, FDA, Bethesda, MD, USA; 3 JH-Institute of Molecular Medicine, Jamia Hamdard, New Delhi, India; 4 Department of Dermatology, VMMC and Safdarjung Hospital, India; 5 Department of Medicine, VMMC & Safdarjung Hospital, New Delhi, India; 6 Department of Biological Sciences, Birla Institute of Technology and Science Pilani, Rajasthan, India

## Abstract

Currently no effective vaccine is available for human visceral leishmaniasis(VL) caused by *Leishmania donovani*. Previously, we showed that centrin1 and p27gene deleted live attenuated *Leishmania* parasites (*Ld*Cen1^−/−^ and *Ld*p27^−/−^) are safe, immunogenic and protective in animal models. Here, to assess the correlates of protection, we evaluated immune responses induced by *Ld*Cen1^−/−^ and *Ld*p27^−/−^ in human blood samples obtained from healthy, healed VL (HVL), post kala-azar dermal leishmaniasis(PKDL) and VL subjects. Both parasites infected human macrophages, as effectively as the wild type parasites. Further, *Ld*Cen1^−/−^ and *Ld*p27^−/−^ strongly stimulated production of pro-inflammatory cytokines including, IL-12, IFN-γ, TNF-α, IL-2, IL-6 and IL-17 in the PBMCs obtained from individuals with a prior exposure to *Leishmania* (HVL and PKDL). There was no significant stimulation of anti-inflammatory cytokines (IL-4 and IL-10). Induction of Th1 biased immune responses was supported by a remarkable increase in IFN-γ secreting CD4^+^ and CD8^+^ T cells and IL-17 secreting CD4^+^ cells in PBMCs from HVL cases with no increase in IL-10 secreting T cells. Hence, *Ld*Cen1^−/−^ and *Ld*p27^−/−^ are promising as live vaccine candidates against VL since they elicit strong protective immune response in human PBMCs from HVL, similar to the wild type parasite infection, mimicking a naturally acquired protection following cure.

Human visceral leishmaniasis (VL) or kala-azar is a potentially fatal disease with an estimated incidence of 0.2 to 0.4 million cases worldwide, causing 20,000–40,000 deaths every year^1^,^2^. Following therapeutic cure of VL, approximately 5–15% cases in India and 50–60% of cases in Sudan develop post kala-azar dermal leishmaniasis (PKDL), which manifests as a dermatitis^3^. PKDL patients are considered to be a major parasite reservoir particularly in India where transmission of VL occurs through anthroponotic route^4^. At present, there is no effective vaccine available to treat or prevent human VL^5^,^6^. The epidemiological observations that individuals recovered from a *Leishmania* infection develop lifelong immunity against reinfection, suggests the possibility of developing a prophylactic vaccine.

Till now in humans the best protection against leishmaniasis has been achieved by leishmanization, which represents inoculation of a low dose of live *Leishmania* promastigotes at the chosen site, usually the arm^7^,^8^,^9^,^10^, however; due to issues related to safety, this practice has been abandoned^11^. Studies have also shown that the limited persistence of parasite is an important factor to develop long lasting immunity^12^,^13^,^14^ and durable protective immunity can be induced by live attenuated parasites^15^,^16^. The major advantage is that these parasites are taken by the host cells similar to virulent parasites, deliver several antigens and do not require adjuvant as compared to the subunit or recombinant vaccine. Live attenuated parasites developed by genetically defined irreversible mutations would be safer compared to the parasite lines that have been developed through other means including long term culture, irradiation or chemical treatment. Limited persistence of the parasites and the immunomodulatory functions exerted on the antigen presenting cells by the attenuated parasites^17^ might help the host to develop a strong memory response.

We have developed two genetically defined live attenuated *Leishmania donovani* parasites, one lacking centrin1, a growth regulating gene (*Ld*cen1^−/−^)^18^ and another lacking p27 gene (*Ldp*27^−/−^)^19^, an essential component of cytochrome c oxidase complex, involved in oxidative phosphorylation. Attenuation of growth and virulence in both *Ld*Cen1^−/−^ and *Ld*p27^−/−^ occur specifically at the intracellular amastigote stage, hence the major advantage is that the parasites can be easily propagated as promastigotes and upon infection of host cells would undergo limited replication as amastigotes without causing pathology. As vaccine candidates, both *Ld*Cen1^−/−^ and *Ld*p27^−/−^ have been found to be safe, immunogenic and protective in various animal models^15^,^16^,^19^,^20^,^21^. Furthermore, both *Ld*Cen1^−/−^ and *Ld*p27^−/−^ induced production of pro-inflammatory cytokines in murine macrophages by classical activation and also skewed antigen presentation abilities of the macrophages more towards a Th1 response that favors development of protective immunity^17^. Lack of knowledge regarding clear biomarkers associated with immunological protection in humans remains a barrier for development of an effective vaccine. Previous studies have shown that *Leishmania* antigens that elicit higher IFN-γ and TNF-α response in healed VL (HVL) as compared to active VL cases have better potential as vaccine candidates^22^ and the vaccine candidates that provide strong protective efficacy in experimental models, generally induce Th1 recall response in PBMCs isolated from HVLcases^23^.

As the animal models do not fully recapitulate the full spectrum of human-parasite interactions, translation of results obtained from studies in the animal models remains a major challenge^24^,^25^. We have undertaken studies using human blood samples obtained from different clinical groups to identify the correlates of protection using two live attenuated parasites (*Ld*Cen1^−/−^ and *Ld*p27^−/−^). Our results demonstrated a strong Th1 response to infection with the live attenuated parasites in the individuals pre-exposed to *Leishmania* parasite. Moreover, the infectivity to macrophages and the immune responses elicited in human PBMCs by the live attenuated parasites was similar to that by the wild type parasite.

## Results

### *Ld*Cen1^−/−^ and *Ld*p27^−/−^ parasites infect human macrophages similar to wild type

**Figure 1:**
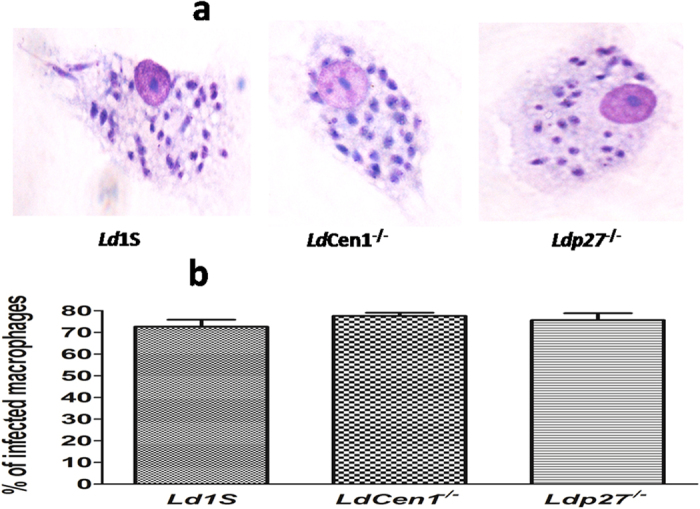
*In vitro* infectivity of live attenuated *Leishmania* parasites. Human PBMCs differentiated macrophages were infected with wild type (*Ld*1S), *Ld*Cen1^−/−^ or *Ld*p27^−/−^ parasites for 6 hours in a ratio of 10:1 (parasites:macrophage). (**a**) Macrophages infected with *Ld*1S, *Ld*Cen1^−/−^ and *Ld*p27^−/−^ respectively after staining with Diff-Quik, (**b**) Percentages of infected macrophages determined by counting a minimum of 300 macrophages per sample under microscope (100X). Results are shown as mean ± SEM for three cover slips for each treatment and are pooled from three different experiments.

Previously we have shown that both *Ld*Cen1^−/−^ and *Ld*p27^−/−^ infect mouse macrophages effectively^15^,^17^,^19^. In the present study, we have determined infectivity of the parasites and compared to the wild type infection, in macrophages obtained by differentiation of human PBMCs. Macrophages were infected with early stationary phase promastigotes at 1:10 macrophage to parasite ratio. Following this protocol, >70% of the population was infected after 6 hours of infection with either *Ld*Cen1^−/−^ or *Ld*p27^−/−^ with an average of 5 to 6 parasites phagocytosed per cell. Further, the percentage of macrophages infected with the live attenuated parasites was similar to that of the wild type (Fig. 1).

### *Ld*Cen1^−/−^ and *Ld*p27^−/−^ strongly induce pro-inflammatory cytokines in PBMCs from healed VL and PKDL subjects

**Figure 2:**
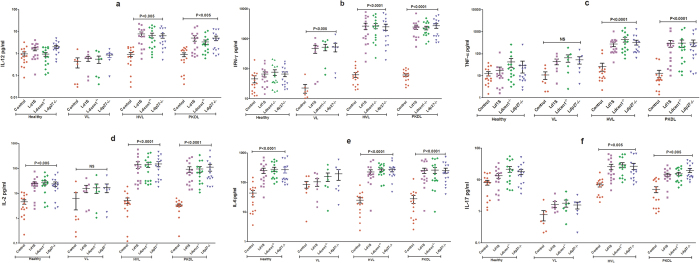
Level of pro-inflammatory cytokines stimulated by wild type (*Ld*1S), *Ld*Cen1^−/−^ and *Ld*p27^−/−^ parasites in culture supernatant of PBMCs obtained from Healthy, HVL, VL and PKDL patients. The results are expressed as scattering of individual values and data are given as Mean ± SEM (pg/ml) of (**a**) IL-12, (**b**) IFN-γ, (**c**) TNF-α, (**d**) IL-2, (**e**) IL-6 and (**f**) IL-17. Significance was determined by Mann-Whitney U test. P < 0.05 is considered statistically significant.

**Figure 3:**
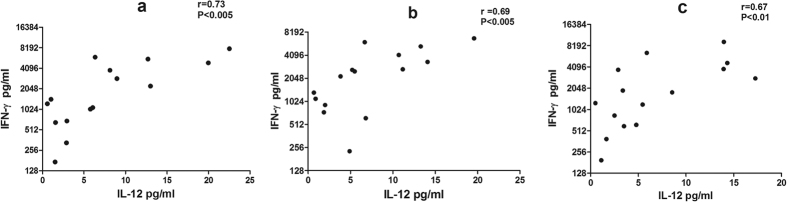
Pairwise correlation of IL-12 and IFN-γ cytokines stimulated in response to (**a**) wild type (*Ld*1S), (**b**) *Ld*Cen1^−/−^ and (**c**) *Ld*p27^−/−^ parasites in culture supernatant of PBMCs obtained from HVL. Significance was determined by Spearman’s rank correlation test. P < 0.05 is considered statistically significant.

Cytokine response to *Ld*Cen1^−/−^ and *Ld*p27^−/−^ was evaluated in the culture supernatant of blood PBMCs of 15 Naïve healthy, 15 HVL, 15 PKDL and 7 active VL patients. TH1 response was evaluated in culture supernatants by measuring level of cytokines IL-12, IFN-γ, TNF-α and IL-2, (Fig. 2). We observed that both *Ld*Cen1^−/−^ and *Ld*p27^−/−^ induced significantly higher levels of IL-12 in the PBMCs from the HVL and PKDL subjects when compared to the control uninfected cells (P < 0.005) (Fig. 2a). No significant stimulation of IL-12 was observed in the VL group or healthy naive subjects (Fig. 2a). Further, both *Ld*Cen1^−/−^ and *Ld*p27^−/−^ stimulated very high levels of IFN-γ in HVL and PKDL as compared to the control uninfected cells (P < 0.0001) (Fig. 2b). In our study we found significantly higher stimulation of IFN-γ in response to *Ld*Cen1^−/−^ and *Ld*p27^−/−^ in the VL group also (P < 0.005), although level of stimulation was lower in comparison to HVL and PKDL (Fig. 2b). Significantly higher stimulation of TNF-α was found only in HVL and PKDL (P < 0.0001) group in comparison to the control uninfected cells (Fig. 2c). Additionally, significantly higher stimulation of IL-2 in response to parasite exposure was observed in HVL, PKDL and healthy subjects; however, level of significance was higher in HVL and PKDL (P < 0.0001) as compared to the healthy subjects (P < 0.005) (Fig. 2d). IL-6 showed significantly higher level of stimulation (P < 0.001) in HVL, PKDL and healthy groups with similar level of stimulation in all the three groups (Fig. 2e). Significantly higher level of stimulation of IL-17 was seen only in HVL and PKDL (P < 0.005) while no stimulation was seen in healthy and VL groups (Fig. 2f). Moreover, stimulation of all cytokines measured in the study showed by both *Ld*Cen1^−/−^ and *Ld*p27^−/−^ parasites was similar to that by the wild type parasites with no significant difference among the attenuated parasite and virulent infections (Fig. 2). As expected, PHA used as a positive control showed very high stimulation as compared to the control unstimulated cells for IFN-γ, TNF-α, IL-6 and IL-17 in PBMCs of all groups (data not shown). Furthermore, the data was compared between different clinical groups and we observed that stimulation in response to *Ld*Cen1^−/−^ and *Ld*p27^−/−^ and wild type parasites in HVL and PKDL groups was significantly higher for IL-12, IL-17, IFN-γ and TNF-α as compared to the healthy group; however, no difference was found in the level of significance for IL-2 and IL-6 (Supplementary Fig. 1). As compared to the VL group stimulation of all cytokines was significantly higher for HVL and PKDL groups. No significant difference in stimulation of cytokines was found between healthy and VL group except for IFN-γ (Supplementary Fig. 1). Of note, we also determined the correlation between different cytokines and found that PBMCs of HVL group showed positive pair wise correlation between the two critical TH1 cytokines i.e. IL-12 and IFN-γ after exposure to *Ld*1S (r = 0.73, P < 0.005), *Ld*Cen1^−/−^ (r = 0.69, P < 0.005) and *Ld*p27^−/−^ (r = 0.67, P < 0.005) (Fig. 3). However, no significant correlation was found among other cytokines (Data not shown).

### *Ld*Cen1^−/−^ and *Ld*p27^−/−^ parasites do not induce disease promoting anti-inflammatory cytokines in PBMCs of any group

**Figure 4:**
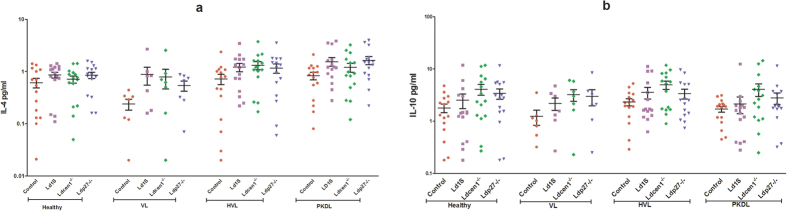
Level of anti-inflammatory cytokines stimulated by wild type (*Ld*1S), *Ld*Cen1^−/−^ and *Ld*p27^−/−^ parasites in culture supernatant of PBMCs obtained from Healthy, HVL, VL and PKDL patients. The results are expressed as scattering of individual values and data are given in Mean ± SEM (pg/ml) of (**a**) IL-4 and (**b**) IL-10. Significance was determined by Mann-Whitney U test. P < 0.05 is considered statistically significant.

Anti-inflammatory response was evaluated by analyzing stimulation of IL-4 and IL-10, both being immunosuppressive cytokines that promote VL pathogenesis. There was no induction in levels of IL-4 or IL-10 in response to *Ld*Cen1^−/−^ or *Ld*p27^−/−^ in any of the groups examined in comparison with the control uninfected cells (P > 0.05), similar to the response to wild type parasite (Fig. 4). Positive control with PHA showed significantly higher stimulation only for IL-10 and this stimulation was found in all the groups including healthy and VL (data not shown). Further, no difference was found in stimulation of IL4 and IL-10 among healthy, VL, HVL and PKDL groups (Supplementary Fig. 2).

### CD4^+^ and CD8^+^ T cells produce IFN-γ and IL-17 in response to live attenuated parasite in HVL group

**Figure 5:**
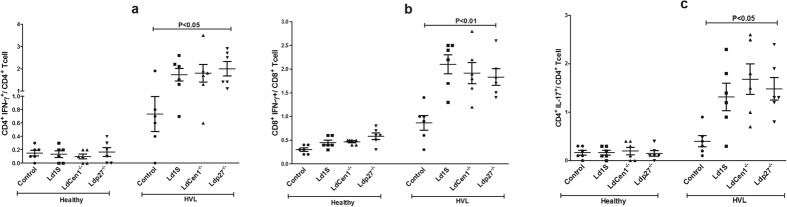
Percentage of IFN-γ and IL-17 -producing cells after stimulation of blood PBMCs obtained from healthy and HVL with wild type (*Ld*1S), *Ld*Cen1^−/−^ and *Ld*p27^−/−^ parasites. The results are expressed as scattering of individual values and data are given in Mean ± SEM (pg/ml) of (**a**) % CD4^+^IFN-γ^+^/CD4^+^ cells, (**b**) % CD8^+^IFN-γ^+^/CD8^+^ cells and (**c**) % CD4^+^IL-17^+^/CD4^+^ cells.

**Figure 6:**
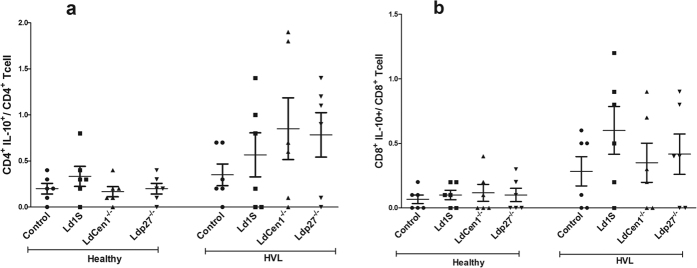
Percentage of IL-10 producing cells after stimulation of blood PBMCs obtained from healthy and HVL with wild type (*Ld*1S), *Ld*Cen1^−/−^ and *Ld*p27^−/−^ parasites. The results are expressed as scattering of individual values and data are given in Mean ± SEM (pg/ml) of (**a**) % CD4^+^IL-10^+^/CD4^+^ cells and (**b**) % CD8^+^IL-10^+^/CD8^+^ cell.

**Figure 7:**
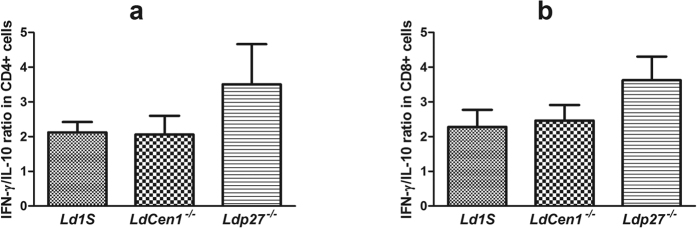
Ratio of IFN-γ/IL-10 producing cells after stimulation of blood PBMCs obtained from HVL with wild type (*Ld*1S), *Ld*Cen1^−/−^ and *Ld*p27^−/−^ parasites. The results are expressed as bar graph and data are given in Mean ± SEM (pg/ml) of (**a**) Ratio IFN-γ/IL-10 producing CD4^+^ cells and (**b**) Ratio IFN-γ/IL-10 producing CD8^+^ cells.

We analyzed the phenotype of cytokine producing cells in response to parasite exposure in healthy and HVL PBMCs. In HVL group, we observed that percentage of IFN-γ producing CD4^+^ and CD8^+^ T cells was significantly increased (P < 0.05) after stimulation with the live attenuated parasites as compared to the control unstimulated cells and the increase in CD8^+^ T cells was more significant (P < 0.01) as compared to the CD4^+^ T cells (P < 0.05). No significant increase was found in the percentage of IFN-γ producing CD4^+^ or CD8^+^ T cells in the healthy group (Fig. 5a,b). We also found significant increase in the percentage of IL-17 producing CD4^+^ T cells after live parasite stimulation in cultures from HVL individuals, in comparison to non-stimulated culture (P < 0.05) (Fig. 5c). Further, we evaluated the effect of parasite exposure on the percentage of IL-10 producing CD4^+^ and CD8^+^ T cells, and found no significant increase in the percentage of IL-10 producing CD4^+^ or CD8^+^ T cells in either healthy or HVL group (P > 0.05) (Fig. 6a,b). An increased ratio of IFN-γ/IL-10 has been correlated with parasite clearance in VL^15^. Parasite exposed PBMCs of HVL group showed an elevated ratio of IFN-γ/IL-10 producing CD4^+^ and CD8^+^ T-cells (Fig. 7a,b), suggesting that exposure of PBMCs to *Ld*Cen1^−/−^ and *Ld*p27^−/−^ live attenuated parasites induced a host-protective cell-mediated pro-inflammatory cytokines producing CD4^+^ and CD8^+^ T cells.

## Discussion

VL is one of the world’s most neglected parasitic diseases second only to malaria^26^. In this study, we report the immune response generated by two distinct live attenuated vaccine candidates (*Ld*cen1^−/−^ and *Ld*p27^−/−^) in human PBMCs obtained from different clinical groups in comparison with the immune response generated by *ex vivo* infection with the wild type parasite.

We found that macrophage infectivity of both *Ld*Cen1^−/−^ and *Ld*p27^−/−^ was comparable to that of the wild type parasite, indicating that the attenuation did not limit the ability to infect human macrophages, consistent with the findings in animal models^15^,^17^,^19^. Previously, we have shown that infection by *Ld*Cen1^−/−^ and *Ld*p27^−/−^ parasites resulted in classical activation of murine macrophages (M1 phenotype) as reflected by increased production of pro-inflammatory cytokines, chemokines, reactive oxygen species, nitric oxide and reduced production of anti-inflammatory cytokines and arginaseactivity^17^. In our study the observed increase in pro-inflammatory cytokines production by *Ld*cen1^−/−^ and *Ld*p27^−/−^ in PBMCs culture from pre-exposed group is consistent with that observed in classically activated murine macrophages.

Studies have shown that the leishmaniasis outcome is mainly determined by Th1/Th2 balance and Th1 response is known to provide protection by an induction of pro-inflammatory response leading to macrophage activation and elimination of intracellular parasite, whereas a Th2 response increases susceptibility to the disease^23^,^27^,^28^. Live attenuated parasites that can predominantly elicit a Th1 response in individuals previously exposed to *Leishmania* infection would be good candidates for prophylactic and/or therapeutic vaccines, as was shown in similar studies with other vaccine formulations including recombinant antigen vaccines^23^. We evaluated pro-inflammatory response by measuring IL-12, IL-2, TNF-α, IFN-γ, IL-6 and IL-17 cytokines and found that all were significantly elevated in HVL and PKDL groups, suggesting that the live attenuated parasite strains induced a Th1 biased immunity.

IL-12 is a critical cytokine that helps in differentiation of naive CD4^+^ T cells into Th1 cells and plays a major immune-regulatory role in the development of cell-mediated immunity (CMI) during intracellular bacterial or parasitic infections by activating macrophages to produce IFN- γ and TNF-α^29^,^30^. Previously, we had shown that the PBMCs of *Ld*Cen1^−/−^ immunized dogs^20^ and *Ld*p27^−/−^ immunized mice^16^ produced elevated level of IL-12 in response to *Leishmania* antigen. Here we found that both *Ld*Cen1^−/−^ and *Ld*p27^−/−^ stimulated significantly higher level of IL-12 in PBMCs from HVL and PKDL cases that are already exposed to *Leishmania* parasite and mimic a naturally protected individual. IL-12 is secreted by antigen presenting cells (APCs) upon activation and dendritic cells serve as primary APCs in the initial phase of *Leishmania* infection^31^. The observed increase in production of IL-12 after infection with *Ld*Cen1^−/−^ and *Ld*p27^−/−^ indicates that the *de novo* antigen presenting function of APC may be fully functional in HVL and PKDL and that unlike virulent parasites, *Ld*Cen1^−/−^ and *Ld*p27^−/−^ parasites do not induce immunesuppressive activities. In contrast, IL-12 production in active VL cases was low suggesting a poor function of dendritic cells in that group. Our studies in mice have shown that the infection of macrophages with *Ld*Cen1^−/−^ or *Ld*p27^−/−^ does not alter the membrane architecture, hence does not affect the antigen presentation ability^17^. However, wild type *Leishmania* parasite depletes membrane cholesterol of macrophages, resulting in defective antigen presentation to T cells^32^. The robust production of IL-12 in our *ex vivo* studies with human PBMCs indicates that the attenuated parasites showed similar macrophage/dendritic cells activation as observed in murine studies. In our previous studies we have shown that both *Ld*Cen1^−/−^ and *Ld*p27^−/−^ can induce IFN-γ production in animal models^15^,^16^,^17^. In the present study, we observed significantly higher stimulation of IFN-γ in HVL and PKDL group as compared to the control uninfected cells and healthy group. Leishmanial antigens have previously been shown to induce stimulation of IFN-γ in PKDL and healed cases of VL, asymptomatic VL, cutaneous and mucocutaneous leishmaniasis^33^,^34^,^35^,^36^,^37^. In the present study significantly higher stimulation of IFN-γ was also found in the VL group as compared to the healthy group. PBMCs from VL cases do not generally produce IFN-γ in response to *Leishmania* antigen^33^,^38^,^39^, however, some studies have shown that human whole blood/PBMCs can indeed produce IFN-γ in active VL^40^,^41^,^42^,^43^. It is important to note that the level of stimulation of IFN-γ observed in the active VL samples were much lower than in HVL and PKDL samples. It is also pertinent to highlight that unlike most previous studies where antigenic re-stimulation was done using soluble *Leishmania* antigen, our study used live parasites (virulent and attenuated) for this purpose suggesting that immunomodulatory activities of the live parasites could be responsible for the observed levels of IFN-γ.

Evaluation of 6 defined *Leishmania* antigen vaccine candidates revealed that only 2 antigens, which were the most immunogenic and protective in murine model, induced IFN-γ production in HVL cases, indicating that *Leishmania* antigens that are protective in experimental models, do not necessarily induce immune response in HVL^23^. Recognition of a single antigen by T cells from individuals with different immunologic and genetic background cannot be always expected, suggesting that the appropriately modified whole parasites would make a better vaccine^23^. IL-12 is a potent inducer of IFN-γ^44^ and our findings also point towards a positive pair wise correlation between IL-12 and IFN-γ. Therefore, it is likely that the increased production of IL-12 observed here also induced production of IFN-γ, which further would activate macrophages for generation of ROS and NO for subsequent killing of intracellular *Leishmania* parasite after a virulent infection^17^. We analyzed the capacity of *Ld*Cen1^−/−^ and *Ld*p27^−/−^ to stimulate TNF-α and found that similar to IL-12, significantly higher stimulation of TNF-α was seen only in the HVL and PKDL groups. The observed increase in production of Th1 cytokines (IFN-γ, TNF-α and IL-12) in *Leishmania* exposed group (HVL or PKDL) compared to naive or active VL individual in response to live attenuated or wild type *Leishmania* parasites, is in accordance with earlier studies carried out with *Leishmania* antigen in HVL and PKDL groups^33^,^34^,^40^,^44^. Further, this increase in Th1 cytokine production in pre-exposed group suggested that the live attenuated parasites can induce protective effector response from memory response generated during resolution of infection in HVL individuals.

Th-17 cells play complementary role along with Th1 to provide protection against VL by producing IL-17 and IL-22 cytokines^45^. IL-17 is a pro-inflammatory cytokine, although little is known about its role during VL infection. Studies have shown that it provides protection against VL^45^ by activating innate immunity and inducing expression of innate inflammatory mediators, including IL-6, GM-CSF, IL-1, IL-8, TNF-α and inducible nitric oxide synthase (iNOS)^46^. Further, it acts synergistically with IFN-γ to strengthen Th1 response and also prevents Treg and IL-10^+^ cell expansion, which helps in controlling parasite replication^47^. In our study, both live attenuated parasites significantly stimulated IL-17 from blood PBMCs of HVL and PKDL groups, which will further promote the leishmanicidal activity of macrophages and improve TH1 response. Anti-inflammatory response induced by *Ld*Cen1^−/−^ and *Ld*p27^−/−^ in PBMCs was evaluated by measuring IL-4 and IL-10. Both IL-4 and IL-10 increase susceptibility for *Leishmania* infection by inducing a Th2 response and are important for the prediction of vaccine success^22^. We observed no significant difference in stimulation of IL-4 and IL-10 between parasite exposed and control-uninfected cells in any study group. Our group has previously shown that, production of IL-4 and IL-10 was not induced in animal models vaccinated with these live attenuated parasites after virulent challenge^15^,^16^,^21^. Previous studies have also shown that PBMCs from cured VL and naïve individual failed to stimulate IL-10 in response to *Leishmania* antigen vaccine candidates^22^,^33^. Importantly, lack of significant stimulation of IL-4 and IL-10 upon infection with *Ld*Cen1^−/−^, *Ld*p27^−/−^ and wild type infection suggests that the two attenuated parasites do not induce a disease promoting immune response significantly different than that of the wild type infection indicating the safety of these attenuated parasites.

In order to assess the intracellular cytokines producing cells after exposure to the parasites, we analyzed the production of IFN-γ, IL-17 and IL-10 by CD4^+^ and CD8^+^ T cells. CD4^+^ cells with the Th1 type cytokine profile such as IFN-γ provide protection against leishmaniasis and it is well established that CD8^+^ T cells play a potential role in the cure of leishmaniasis, particularly VL by exerting its cytotoxic effect^33^,^42^. We found that PBMCs infected with live attenuated and wild type parasite displayed higher frequency of CD4^+^ T cells expressing IL-17 and both CD4^+^ as well as CD8^+^ T cells expressing IFN-γ in HVL, while no significant increase was seen in IL-10 secreting cells. This suggests that similar to a naturally occurring exposure to virulent parasites of the HVL individuals following cure in *Leishmania* endemic areas, *ex vivo* infection with *Ld*Cen1^−/−^ and *Ld*p27^−/−^ also produced an immune response consistent with a protection outcome. Similar results were observed in mice and dogs immunized with these live attenuated parasites where stimulation was found in *Leishmania* antigen experienced IFN-γ secreting effector CD4^+^ and CD8^+^ T cells but not in IL-10 secreting cells^15^,^16^,^20^. Previous studies with human subjects have also shown that the increase in IFN-γ producing CD4^+^ and CD8^+^ T cells in response to *Leishmania* antigen was stimulated mainly in PBMCs of individuals cured of VL^42^,^48^ and CL^49^ infection. CD8^+^ T-cells were shown to be exhausted in VL cases hence failed to produce IFN-γ in response to *Leishmania* antigen in whole blood cultures; however, the ability of CD8^+^ T-cells to produce antigen specific IFN-γ was restored following clinical cure (HVL)^42^. Another study with whole *Leishmania* parasite (live/dead) showed that proliferation of CD4^+^ and CD8^+^ T cells was significantly higher in cured CL individuals as compared to the healthy individuals. Further, the stimulation of IFN-γ secreting CD4^+^ and CD8^+^ T cells was higher with live attenuated parasites as compared to the killed parasites, providing another evidence that live attenuated *Leishmania* parasites are better in inducing protective immune response as compared to the killed parasites^50^. This increase in percentage of pro-inflammatory cytokine producing CD4^+^ and CD8^+^ T cells upon stimulation with live attenuated parasites in HVL group corroborates with our cytokine profile data observed in PBMCs culture supernatants. As IFN-γ and IL-10 are the two main regulatory cytokines governing the fate of the infection in VL, their ratio has been correlated to parasites elimination and indicator of vaccine success^51^. The increased ratio of IFN-γ: IL-10 producing CD4^+^ and CD8^+^ T cells in HVL after infection with *Ld*Cen1^−/−^ and *Ld*p27^−/−^ provide another correlate of protection. Previously, we have shown similar polarization to increased ratio of IFN-γ: IL-10 producing CD4^+^ and CD8^+^ T cells after parasite challenge in mice immunized with *Ld*Cen1^−/−^ and *Ld*p27^−/− ^15,16.

One of the major challenges for elimination of VL is the presence of asymptomatic carriers that are difficult to identify and might serve as a parasite reservoir^52^. Asymptomatic individuals reach a state of acquired protection against leishmaniasis due to low level parasite infection as suggested by earlier studies^53^. The nature and durability of this protection is indeterminate, however, it is reasonable to argue that vaccination of asymptomatic carriers with live attenuated parasites would be beneficial in maintaining or perhaps adding to the protective immunity due to a favorable immune environment induced by the previous exposure to low level of virulent parasites. Previous analyses have indicated that in the absence of drug treatment, vaccination of asymptomatic carriers presents the best approach to prevent and eliminate VL^54^,^55^.

In summary, this study evaluated the capacity of *Ld*Cen1^−/−^ and *Ld*p27^−/−^ live attenuated vaccine candidates to elicit immune responses in PMBCs obtained from individuals with distinct clinical status, including naïve healthy, active VL, HVL and PKDL groups. Previously, we have established that *Ld*Cen1^−/−^ and *Ld*p27^−/−^ are immunogenic, protective and safe in animal models. The present work is a step forward, demonstrating that *Ld*Cen1^−/−^ and *Ld*p27^−/−^ are safe, immunogenic and induce protective immune response in human PBMCs comparable to the wild type parasite, therefore both have great potential as live attenuated vaccine against VL.

## Material and Methods

### Study subjects and ethical consideration

Study was carried out in 4 groups; Naïve healthy (n = 15), Healed VL (n = 15), PKDL (n = 15) and active VL (n = 7) patients. Individuals unexposed to *Leishmania* parasite i.e. living in VL non-endemic areas and negative for rk39 strip test were included in the naïve healthy group while those having a previous history of VL that was cured at least 1 year before the recruitment in the study were included in the HVL group. Patients reported at the Safdarjung hospital, New Delhi, showing clinical symptom of the disease and positive for PCR/ LD bodies or both in tissue biopsy/BMA were included in PKDL and VL group. Naïve healthy individuals were included because they are never exposed to *Leishmania donovani* and serve as negative controls while HVL and PKDL cases are already exposed and are presumably immune to recurrence of VL. The study was approved by and carried out under the guidelines of the Ethical Committee of the Safdarjung Hospital, India. Written informed consent for participation was obtained from all participants including the healthy subjects.

### Parasite culture

*Leishmania donovani* promastigotes were cultured according to the procedure previously described^56^,^57^. In brief, wild type *Ld*1S was cultured in M199 medium containing 10% heat inactivated fetal bovine serum while *Ld*Cen1^−/−^ and *Ld*p27^−/−^ were grown in the same medium with added antibiotics; hygromycin (40 μg/ml) and G418 (40 μg/ml) for *Ld*Cen1^−/−^ and G418 (40 μg/ml) for *Ld*p27^−/−^. All parasite cultures were incubated at 26 °C in a BOD incubator and early stationary phase promastigotes were used in the study.

### PBMCs isolation and stimulation with parasites

Heparinized blood was collected from all the study subjects and PBMCs were isolated from blood by density gradient centrifugation with Ficoll-Hypaque (Sigma-Aldrich) method. The cells were cultured in RPMI 1640 supplemented with 10% FCS, glutamine, HEPES and penicillin (100 U/ml), and streptomycin (100 μg/ml). 2 × 10^5^ PBMCs were plated in each well of 96 well flat bottom tissue culture plates and incubated in triplicate with (i) only media as unstimulated control, (ii) PHA (1 μg ⁄mL) as a positive control, (iii) 1 × 10^4^ live wild type *L. donovani* (*Ld*1S), (iv) 1 × 10^4^ live *Ld*Cen1^−/−^ or (v) 1 × 10^4^ live *Ld*p27^−/−^ parasites. All parasite cultures were washed three times with PBS before incubation with PBMCs. After 120 hours of incubation in a 5% CO_2_ humidified atmosphere at 37 °C, supernatants were collected and stored at −70 °C until further analysis.

### Multiplex ELISA for cytokine estimation

Cytokine levels in PBMC culture supernatants were determined using Human Cytokines, Bio-PlexPro^TM^ (Bio-Rad) kit according to the manufacturer’s protocol. Briefly, a 50 μl cell supernatant sample was incubated with antibody coupled beads. Immune complexes were washed, incubated with biotinylated detection antibody and finally, with streptavidin-phycoerythrin prior to assessing cytokine concentration titers. Manufacturer provided standards were used to prepare the standard curve for each cytokine. A total of 8 human cytokines representing either a pro-inflammatory (IL12, IL2, TNF-α, IFN-γ, IL6 and IL17) or an anti-inflammatory (IL4 and IL10) immune response were analyzed. Cytokine levels were determined using a multiplex array reader from Luminex™ Instrumentation System (Bio-Plex Workstation from Bio-Rad Laboratories). The cytokines concentration was calculated using software provided by the manufacturer (Bio-Plex Manager Software).

### Preparation of human peripheral blood macrophages and *in vitro* macrophage infections

Infectivity of WT, *Ld*Cen1^−/−^ and *Ld*p27^−/−^ parasites was investigated using human PBMCs derived macrophages. PBMCs were isolated from the healthy blood sample as described above and plated in 24 well (1.5 × 10^6^/well) plate containing lysine coated cover slips and incubated for adherence at 37 °C with 5% CO_2_ for 4 hours in RPMI 1640 with 10% FCS. After removing the non-adherent cells by washing with PBS, the plate was further incubated for 8 days in complete RPMI medium supplemented with human MCSF (50 ng/ml) for differentiation of macrophages. After every 48 hours’ cells were washed and fresh medium was added. On day-8 macrophages were infected with early stationary phase parasites at 1:10 macrophage to parasite ratio. After 6 hours of infection, cells were washed to remove non-internalized parasites. Cells adhered to cover slips were fixed in 100% methanol and stained with Diff Quik staining. Percentages of infected macrophages were determined by counting a minimum of 300 macrophages per sample under microscope (100X). Macrophage infectivity assay was performed in three technical and biological replicates for each parasite.

### Intracellular staining and flow cytometry

To determine the cellular source/s and levels of cytokines, flow cytometry was performed with the blood PBMCs obtained from naïve (n = 6) and HVL (n = 6) individuals. PBMCs were stimulated with parasites as described above. Phorbolmyristate acetate (PMA) (50 ng/ml; Sigma-Aldrich) and ionomycin (500 ng/ml; Sigma-Aldrich) were added for 6 hours in PBMCs culture and used as positive controls. To block cytokine secretion, cultures were treated with GolgiStop (1 ug/ml, BD Biosciences) and further incubated for an additional 4 hours. After incubation, cells were washed and surface stained with the following antibodies: CD3-PerCPcy5.5, CD4-PE-CF594 and CD8-APC-H7 for 20 minutes at 4 °C. Surface stained cells were fixed and permeabilized using BD Cytoperm/cytofix kit (BD Biosciences) according to manufacturer’s instructions, washed in permeabilization buffer (BD) and stained for the presence of intracellular IFN-γ, IL-17 and IL-10 using PE-Cy7, Alexa Fluor^®^ 647 and PE conjugated antibodies (BD) respectively. Isotype matched control antibodies were used to detect non-specific binding to the cells. Following intra cellular staining, samples were acquired on FACS Aria and analyzed using FACS Diva software(BD Biosciences). 7AAD staining (BD Biosciences) of a limited number of samples confirmed that the gated lymphocytes were >95% viable for both healthy and HVL group stimulated with parasite. Lymphocytes were gated on the basis of forward and side scatter. From these lymphocyte population CD3^+^ T-cells were gated to determine frequencies of CD4^+^ and CD8^+^ T-cells and for further analysis of IFN-γ, IL-10 and IL-17 expression.

### Statistical analysis

Statistical analysis was performed using Graph Pad Prism 5 software (San Diego, USA). Statistical significance was determined by Mann-Whitney U test. Correlation was evaluated using Spearman’s rank correlation test. P values of less than 0.05 were considered significant.

## Additional Information

**How to cite this article**: Avishek, K. *et al*. Gene deleted live attenuated *Leishmania* vaccine candidates against visceral leishmaniasis elicit pro-inflammatory cytokines response in human PBMCs. *Sci. Rep.* **6**, 33059; doi: 10.1038/srep33059 (2016).

## Supplementary Material

Supplementary Information
