# Supplementary material for: Gene deleted live attenuated Leishmania vaccine candidates against visceral leishmaniasis elicit pro-inflammatory cytokines response in human PBMCs
Source: Sci Rep. 2016 Sep 14;6:33059. doi: 10.1038/srep33059 (PMC5021981; doi:10.1038/srep33059)
Supplement: Supplementary Information [file srep33059-s1.pdf]

Gene deleted live attenuated *Leishmania* vaccine candidates against visceral leishmaniasis elicit pro-inflammatory cytokines response in human PBMCs

Kumar Avishek[1](M.Sc.),Himanshu Kaushal[1](M.Sc.), Sreenivas Gannavaram[2](Ph.D.), Ranadhir Dey[2](Ph.D.), Angamuthu Selvapandiyan[3](Ph.D.), V Ramesh[4](M.D.), Narender Singh Negi[5](M.D.), Uma S. Dubey[6](Ph.D.), Hira L. Nakhasi[2](Ph.D.) and Poonam Salotra[1*](Ph.D.)

[1]National Institute of Pathology (ICMR), Safdarjung Hospital Campus, New Delhi, India.[2]Division of Emerging and Transfusion Transmitted Diseases, CBER, FDA, Bethesda, MD, USA.[3]Institute of Molecular Medicine, New Delhi, India, [4]Department of Dermatology, VMMC and Safdarjung Hospital, India.[5]Department of Medicine, VMMC & Safdarjung Hospital, New Delhi, India. [6]Department of Biological Sciences, Birla Institute of Technology and Science Pilani, Rajasthan, India.

*Reprints or correspondence:

Poonam Salotra
National Institute of Pathology (ICMR)
Safdarjung Hospital Campus
New Delhi-110029, INDIA
Tel: 91-11-2616 6124
Fax: 91-11-2619 8401
E-mail: salotra@vsnl.com

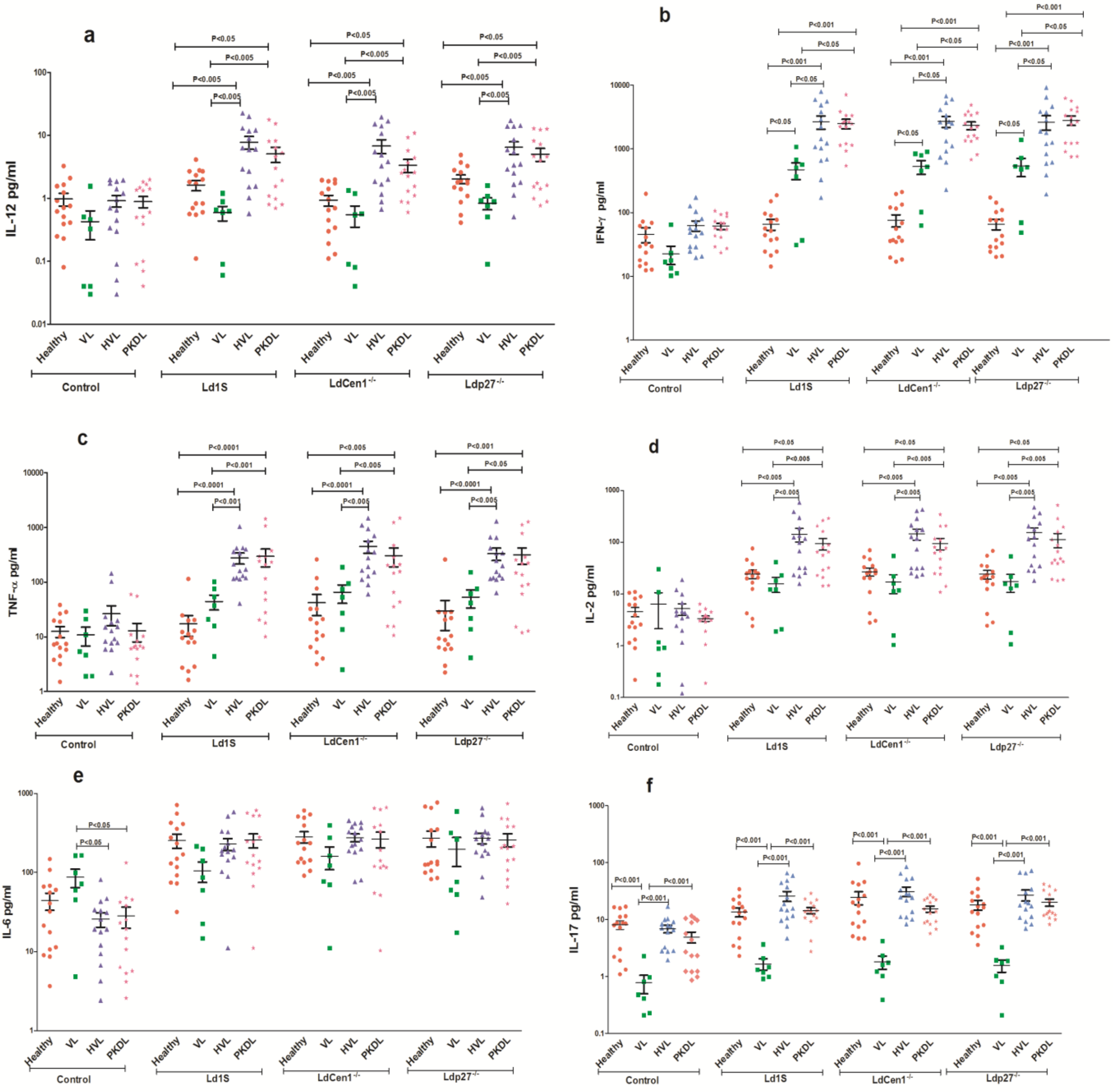

Supplementary figure. 1 Level of pro-inflammatory cytokines stimulated in culture supernatant of PBMCs obtained from healthy, HVL, VL and PKDL groups by wild type (*Ld*1S), *Ld*Cen1$^{-/-}$ and *Ld*p27$^{-/-}$parasites. The results are expressed as scattering of individual values and data are given in Mean±SEM (pg/ml) of (a) IL-12, (b) IFN-γ, (c) TNF- α, (d) IL-2, (e) IL-6 and (f) IL-17. Significance was determined by Mann-Whitney U test. $P < 0.05$ is considered statistically significant.

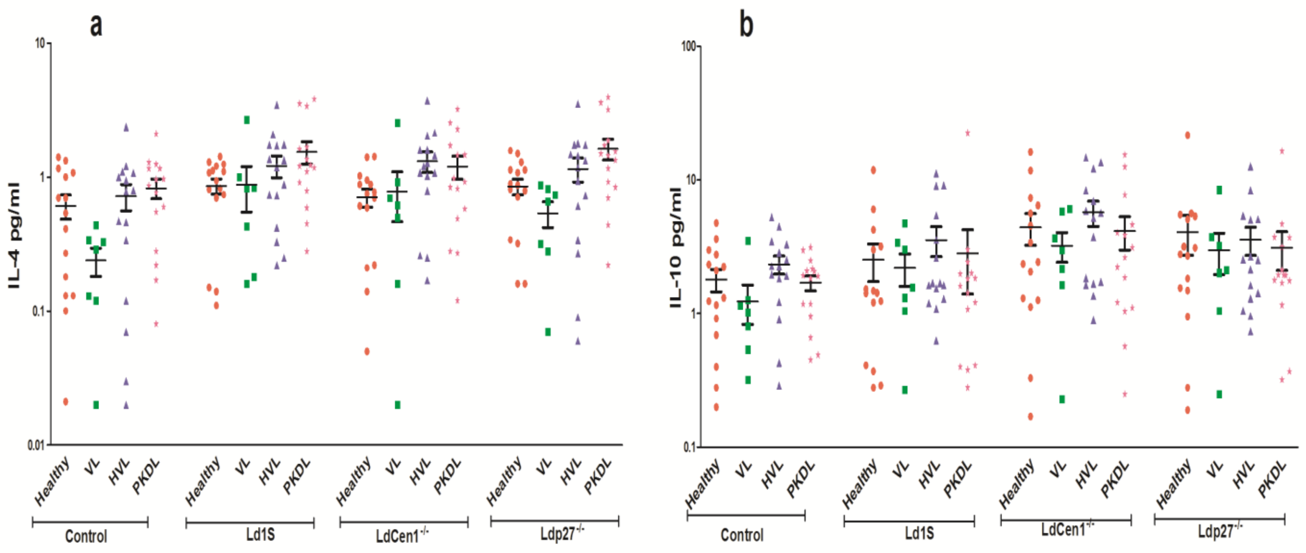

Supplementary figure. 2 Level of anti-inflammatory cytokines stimulated in culture supernatant of PBMCs obtained from healthy, HVL, VL and PKDL groups by wild type (*Ld*1S), *Ld*Cen1$^{-/-}$ and *Ld*p27$^{-/-}$parasites. The results are expressed as scattering of individual values and data are given in Mean±SEM (pg/ml) of (a) IL-4 and (b) IL-10. Significance was determined by Mann-Whitney U test. $P < 0.05$ is considered statistically significant.
